# Conspectus of Organic Materia Medica and Pharmacal Botany

**Published:** 1879-01

**Authors:** 


CONSPECTUS OF ORGANIC MATERIA MEDICA AND PHARMACAL BOTANY, Comprisiug the Vegetable and Animal Drugs: their Physical Character, Geographical Origin, Classification, Constituents, Doses, Adulterations, etc. Table of the Tests and Solubilities of the Alkaloids appended. By L. E. Sayre, Ph. G. Philadelphia: D. G. Brinton, 115 S. Seventh street: 1879.

To the medical botanist this work must prove eminently acceptable. Not intended as a text-book on general materia medica, the author's object seems to be more especially to give the botanical origin of vegetable drugs. Hence, the work is commenced with a chart, giving the name of the natural order, the officinal name, the botanical name, the common name, the habitat, the part used, the constituents, the medical properties, dose and officinal preparations. Then, after thirty pages on general botany, each article of drugs is considered separately, in regard to nativity, characteristics, constituents, adulterations, etc., and it closes with a chart of solubilities and tests.

Taken all together the work will be found interesting and instructive to the medical reader.

